# Glibenclamide Increases Nitric Oxide Levels and Decreases Oxidative Stress in an In Vitro Model of Preeclampsia

**DOI:** 10.3390/antiox11081620

**Published:** 2022-08-20

**Authors:** Priscila Rezeck Nunes, Thaina Omia Bueno Pereira, Mariana Bertozzi Matheus, Nubia Alves Grandini, Juliana Silva Siqueira, Camila Renata Correa, Joelcio Francisco Abbade, Valeria Cristina Sandrim

**Affiliations:** 1 Department of Biophysics and Pharmacology, Institute of Biosciences, Sao Paulo State University (Unesp), Sao Paulo 18618-689, Brazil; 2 Department of Pathology, Medical School, Sao Paulo State University (Unesp), Sao Paulo 18618-687, Brazil; 3 Department of Gynecology and Obstetrics, Medical School, Sao Paulo State University (Unesp), Sao Paulo 18618-687, Brazil

**Keywords:** glibenclamide, preeclampsia, nitric oxide, reactive oxygen species, antioxidant capacity, HUVEC

## Abstract

(1) Background: The bioavailability of nitric oxide (NO) and oxidative stress are important events related to the pathophysiology of preeclampsia (PE). In this present study, we aimed to evaluate the antioxidant effect of glibenclamide (GB) on the NO synthesis, oxidative stress, and antioxidant capacity in endothelial cells incubated with plasma from preeclamptic (PE) and normotensive pregnant women (NT). (2) Methods: Human umbilical vein endothelial cells (HUVECs) were incubated with a plasma pool from 10 NT and 10 PE pregnant women; NO/NOx quantification and ROS levels were assessed by a fluorescence compound; lipid peroxidation was evaluated employing thiobarbituric acid (TBA); and total antioxidant capacity was measured by ferric reduction ability power (FRAP) and 3-(4,5-dimethylthiazol-2-yl)-2,5-diphenyltetrazolium bromide (MTT). (3) Results: We found that endothelial cells incubated with plasma from PE showed lower NO and NOx levels compared with the NT group. However, GB treatment increased these levels, as well as the antioxidant capacity. Furthermore, a decrease was observed in ROS generation and lipid peroxidation (4) Conclusions: The GB treatment exerted a positive effect on the NO/NOx production by HUVEC incubated with plasma from NT and PE pregnant women, as well as in the reduction in oxidative stress and increase in the antioxidant capacity.

## 1. Introduction

Preeclampsia (PE) is a pathology of human pregnancy, which is listed as a syndrome responsible for 2 to 8% of the morbidity and mortality causes of pregnancies worldwide [1]. The initial clinical symptoms considered parameters for the identification of this pathology are arterial hypertension and proteinuria from the 20th week of pregnancy or in the first days after delivery. Recently, other maternal dysfunction may also be related to PE, such as impaired renal function, liver involvement, neurological complications, uteroplacental dysfunction, or fetal growth restriction [1]. It is well-established that placental ischemia plays a fundamental role in the pathophysiology of PE, since this process contributes to the release of products resulting from poor perfusion into the maternal circulation, leading to systemic endothelial dysfunction [2]. Furthermore, PE pregnancies are also characterized by trophoblast immaturity [3], which adds to the systemic endothelial dysfunction and are two very important features that can contribute to the alteration of oxidative stress biomarkers in this disease.

Several studies suggest that the generalized endothelial dysfunction characteristic of PE pregnancies is the main cause of the clinical abnormalities observed in this pathology [4,5]. Endothelial dysfunction could alter the balance between vasoactive substances, such as nitric oxide (NO), prostacyclin, and endothelin [6]. NO plays a key role in endothelium hemostasis since impairments in its production or activity could be associated with the main mechanism that leads to endothelial dysfunction. In the same way, the imbalance between vasoactive factors could lead to failure in controlling vascular tone, and endothelial dysfunction could be associated with an abnormal release of these endothelium-derived factors [7]. The decrease in NO bioavailability (due to decreased synthesis or increased degradation) is intimately linked with endothelial dysfunction, and changes in NO metabolism could be a risk factor to develop PE [8].

Taken together, oxidative stress and inflammation are summation events in inflammatory diseases and both play a pivotal role in PE pathogenesis. The activation of inflammatory cascades can occur due to several stimuli, such as the production of reactive oxygen species (ROS) [9], which are the first reactive intermediates produced during this process, being responsible for the release of inflammatory agents during the immune response [10]. Furthermore, excess production of ROS can increase NO catabolism, leading to a decrease in its bioavailability. This imbalance between ROS-NO leads to the expression of inflammation-related genes, which in turn can impair endothelial function [11].

Therefore, some strategies can be used to ameliorate the inflammatory condition generated in PE. In this sense, some drugs have been tested, such as glibenclamide (GB), a sulfonylurea family drug commonly prescribed to treat type 2 diabetes mellitus. Acting as an anti-inflammatory agent, GB effectively inhibits inflammatory cell migration as it prevents the inflammasome setting (an intracellular multiprotein that activates caspase-1, producing active interleukins (IL)-1β and IL-18 when stimulated properly) [12]. Specifically, GB inhibits inflammasome activation by inducing the closure of adenosine triphosphate (ATP)-sensitive potassium channels, increasing intracellular potassium concentration [13]. In this sense, there is a reduction in inflammatory cell infiltration, preventing damage to organs in ischemic tissue [14,15]. Acting on endothelial dysfunction, GB has been described as an inhibitor of the inflammasome in endothelial cells in the blood–brain barrier [16]. Zhang et al. showed that GB protects from injuries associated with inflammation in vitro and in vivo by the inhibition of inflammasome domains, with consequently decreased the production of pro-inflammatory cytokines, ROS, and migration of inflammatory cells [17]. Other authors showed that GB acts as an anti-inflammatory and decreases ROS production when administrated in a preeclamptic animal model [18].

Regarding GB as treatment for pregnancy-related disorders, some studies have explored the use of GB compared to insulin. Langer et al. showed that GB and insulin have the same efficiency for the treatment of gestational diabetes mellitus (GDM) in all levels of disease severity [19]. Additionally, Leung et al. showed that GB use does not increase the incidence of macrosomia or hypoglycemia when compared with insulin use in patients with GDM. Furthermore, overall pregnancies treated with GB are not at higher risk for adverse neonatal and maternal outcomes compared to those pregnancies treated with insulin [20].

Given that the anti-inflammatory and antioxidant effects of GB in pregnancy are already better explored, this study aimed to evaluate the action of GB in the production of NO and ROS, as well as in the antioxidant capacity of endothelial cells cultured with the PE plasma, allowing the comprehension of molecular mechanisms regarding endothelial dysfunction and oxidative stress in this syndrome.

## 2. Materials and Methods

### 2.1. Patients

For the pool of plasma used in the incubation with HUVECs, 20 pregnant women were selected, distributed as follows: 10 PE and 10 NT. The pregnant women included in the study underwent delivery care at the Maternity Hospital of the Hospital das Clínicas of the Faculty of Medicine of Botucatu—UNESP. All pregnant women signed the Free and Informed Consent Form and the study was approved by the Research Ethics Committee of the Botucatu School of Medicine (nº 4961945, approved on 9 September 2021). The gestational age of the studied groups was established by the date of the last menstrual period and/or confirmed by an early ultrasound examination (<12 weeks of gestation). The diagnosis of PE was made considering when, without a history, a pregnant woman developed hypertension (blood pressure ≥ 140/90 mmHg) with or without proteinuria (≥300 mg in 24-h urine) after the 20th week of gestation. The NT pregnant women did not present hypertensive disorders in pregnancy and remained normotensive until the time of delivery [1].

### 2.2. Plasma Collection and Processing

Peripheral blood from the pregnant women (20 mL) was collected by venipuncture in a sterile tube containing 158 units of Sodium Heparin (BD Vacutainer, Franklin Lakes, NJ, USA) to store the plasma of pregnant women with PE at the time of diagnosis of the disease. Peripheral blood was centrifuged and the plasma obtained was stored at −80 °C until incubation with endothelial cells.

### 2.3. Culture of HUVEC

Human Umbilical Vein Endothelial Cells (HUVEC—EA.hy 926) were acquired with certification that they are cells of this designated lineage. Cells were cultured at 37 °C in 5% CO_2_ in DMEM (Gibco, Waltham, MA, USA) supplemented with 10% (*v*/*v*) fetal bovine serum (FBS), 100 u/mL penicillin, 100 μg/mL streptomycin, and 2 mMol/L L-Glutamine (Sigma-Aldrich, San Luis, MO, USA) until reaching 80–90% of confluence. For the time of the experiments, the cells were incubated at 37 °C in 5% CO_2_ in a medium without FBS with previously standardized concentrations of GB (50,100 and 200 µM—Sigma-Aldrich) for 30 min, and then the pool of plasma (20% *v*/*v*) for 24 h. All experiments were performed using cells until the 10th passage.

### 2.4. Cell Viability Analysis of HUVECs against Incubation with Plasma and Glibenclamide

The cell viability assay was performed using the PrestoBlue^®^ Cell Viability Reagent (Invitrogen) following the manufacturer’s instructions. Procedures were performed in 96-well plates including 5 replicates from each group. To perform the assay, 110 μL of the supernatant was discarded and then 10 μL of PrestoBlue^®^ reagent was added, followed by incubation for 1 h. Cell viability was quantified by fluorescence in a reader.

### 2.5. Evaluation of Cellular Production of Nitric Oxide (NO) and Nitrite/Nitrate (Total NO_x_) in the Cell Culture Supernatant

Cellular quantification of NO was evaluated using the DAF-FM (4-Amino-5-Methylamino-2′,7′-Difluorofluorescein Diacetate) probe (5 µM) (Invitrogen, Thermo Fisher Scientific, Carlsbad, CA, USA). The assay was performed according to the manufacturer’s instructions. The fluorescence signal was measured (excitation 495 nm, emission 535 nm) in a multifunctional plate reader (Synergy 4, BioTek, Winooski, VT, USA).

NOx levels were assessed in HUVEC culture supernatant in triplicate using Griess reagents [21]. Firstly, 50 µL of samples were incubated with 50 µL of 1% sulfanilamide solution in 5% phosphoric acid for 10 min protected from light. Then, 50 µL of 0.1% N-(1-Naphthyl)-ethylenediamine dihydrochloride solution was added, followed by a 10-min incubation. A 96-well plate was read in a spectrophotometer (Synergy 4, BioTek, Winooski, VT, USA,) at 535 nm. A standard curve was generated by incubation of nitrite solutions (1.56–100 µmol/L) with the previous reagents. NOx levels in HUVECs supernatant were expressed in µmol/L.

### 2.6. Reactive Oxygen Species (ROS)

ROS was quantified using 2′-7′-dichlorodihydrofluorescein diacetate—DCFH-DA (Sigma-Aldrich) by fluorescence with 2V, 7V-dichlorofluorescein diacetate. We used 400µM angiotensin II (Sigma-Aldrich) as an inductor of ROS generation, and 100 µM apocynin (Sigma-Aldrich) as an inhibitor of ROS production, incubated 30 min before the pool. The assay was performed following the manufacturer’s instructions.

### 2.7. Assessment of Lipid Peroxidation in Supernatants

Malondialdehyde (MDA) levels were quantified using thiobarbituric acid (TBA) 0.67% (1:1). The acid was added to the supernatant, and then the samples were heated for 45 min in a water bath at 100 °C. MDA was reacted with TBA in a 1:2 MDA-TBA ratio and then read at 535 nm on a Spectra Max 190 microplate reader (Molecular Devices^®^, Sunnyvale, CA, USA). The concentration of MDA was obtained through the molar extinction coefficient (1.56 × 10^5^ M^−1^ cm^−1^) and the absorbances of the samples and the final result was expressed in nmol/g of protein [22].

### 2.8. Antioxidant Capacity

The total antioxidant capacity was performed using the iron reduction assay (Ferric Reducing Antioxidant Power—FRAP), based on the rapid reduction of iron in ferric tripyridyl triazine (FeIII-TPTZ) by antioxidants present in the samples, forming ferrous tripyridyl triazine (FeII- TPTZa), a substance with an intense blue color [23]. Briefly, the working reagent was prepared using 300 mmol/L of acetate buffer, 10mmol/L of TPTZ/HCl solution, and 20 mmol/L of ferric chloride. In a 96-well plate, 10 μL of the sample was added with 290 μL of the working solution. A ferrous sulfate curve ranging from 0.0312 to 4 mmol was constructed and the plate was incubated for 5 min. The absorbance was then read at 593 nm on the spectrophotometer (Synergy 4, Biotek). Data are expressed in μmol/L.

To measure the direct reduction of 3-(4,5-dimethylthiazol-2-yl)-2,5-diphenyltetrazolium bromide—MTT (Sigma-Aldrich), 100 mL of cell supernatant were mixed with 12.5 mL of dye solution (5 mg/mL) in PBS). The final volume was adjusted to 200 mL with PBS and the mixture was incubated for 60 min at 37 °C. The reaction was interrupted by the addition of 750 mL of 0.04 M hydrochloric acid in isopropanol. The tubes were centrifuged for 10 min at 1000× *g*, the supernatant was collected and the absorbance was measured at 570 nm on the spectrophotometer (Synergy 4, Biotek).

### 2.9. Statistical Analysis

Replicates of 5 per group combined with treatments (plasma, GB, inhibitors, and activators) were performed in each experiment. Three independent experiments were performed. When we compare three or more groups, we used One-Way ANOVA followed by the Tukey test. Grouped analyses were performed using Two-way ANOVA followed by Bonferroni’s Multiple Comparison Test. To compare the two groups, we employed the Mann–Whitney U test. Results are expressed in means ± SEM. Statistical analyses were performed using GraphPad Prism 8.0 (GraphPad Software, San Diego, CA, USA) and for all tests, we considered a *p*-value ≤ 0.05 (two-tailed) significant. 

## 3. Results

### 3.1. Clinical Parameters of Patients Used to Constitute Pooled Plasma

Table 1 presents the clinical and biochemical data of study subjects used to constitute the pooled plasma.

No differences were found in the maternal age parameter between the groups. As expected, systolic blood pressure (SBP) and diastolic blood pressure (DBP) are increased in the PE group when compared to the NT group (<0.0001). Gestational age at sampling was lower in the PE group compared to NT (0.0001). The presence of 24 h proteinuria and high uric acid levels are also clinical features that characterize a pregnancy complicated by PE.

### 3.2. HUVEC Incubated with Plasma and Glibenclamide did Not Show Differences in Viability

**Figure 1:**
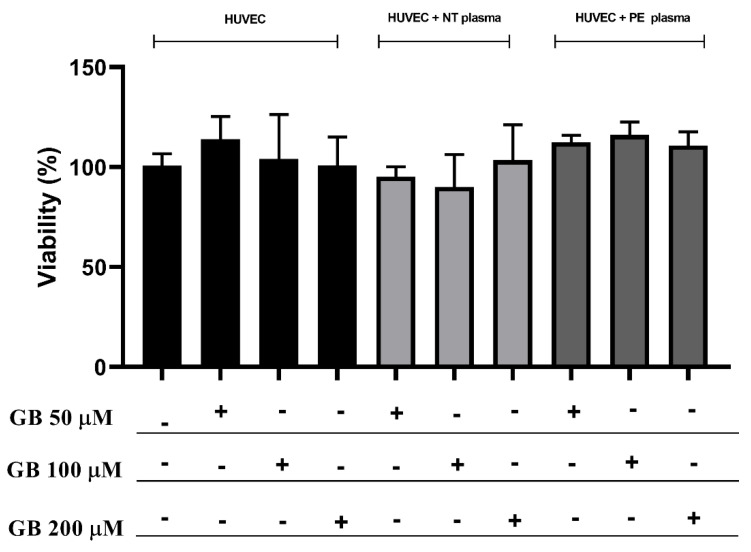
There were no differences in HUVEC viability between treatment with glibenclamide (GB) or the combination of GB (50, 100, and 200 µM) plus plasma of NT (n = 10) and PE (n = 10) pregnant women for 24 h. Three independent experiments with replicates of 5 per group in each experiment. Data are presented as mean ± SD (ANOVA).

Figure 1 shows the viability assay of HUVECs cultured with or without GB and plasma plus GB for 24 h. There is no difference observed in the groups, and all cells treated showed at least 90% viability. The concentration chosen for the following experiments was 100 µM.

### 3.3. GB Increases NO Production in HUVEC Incubated with Plasma from PE and NT Pregnant Women and Nox in Cell Supernatant

**Figure 2:**
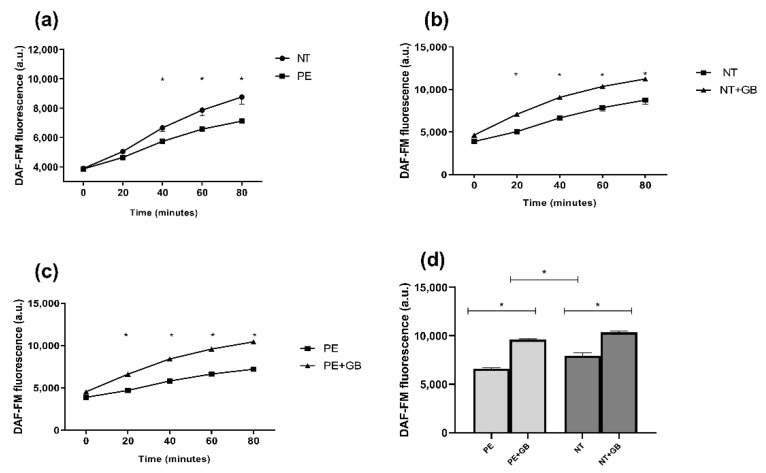
Nitric oxide (NO) fluorescence intensity was measured by DAF-FM for 80 min. Human umbilical vein endothelial cells (HUVECs) were incubated with 20% (*v*/*v*) pooled plasma from NT (n = 10) and PE (n = 10) (**a**) and then plasma plus GB (100 µM) (**b**,**c**) for 24 h. Sixty minutes of incubation with GB and plasma are shown in (**d**). Values are represented as means ± SEM. Comparisons between groups were assessed by Two-Way ANOVA followed by Bonferroni’s Multiple Comparison Test (**a**–**c**) and One-Way ANOVA (**d**) followed by Tukey’s Test. * (*p* < 0.05).

Endothelial cells incubated with a pool of NT plasma induce higher NO levels when compared with the PE group (*p* < 0.05) after 40 min of incubation (Figure 2a). In this group, GB treatment increased NO levels (*p* < 0.05) after 20 min of incubation (Figure 2b) compared with cells without treatment. The same occurred with NO production by cells treated with PE plasma plus GB (Figure 2c). Figure 2d shows NO production in 60 min of incubation with DAF, with differences (*p* < 0.05) between PE versus NT, and PE/NT plus GB.

**Figure 3:**
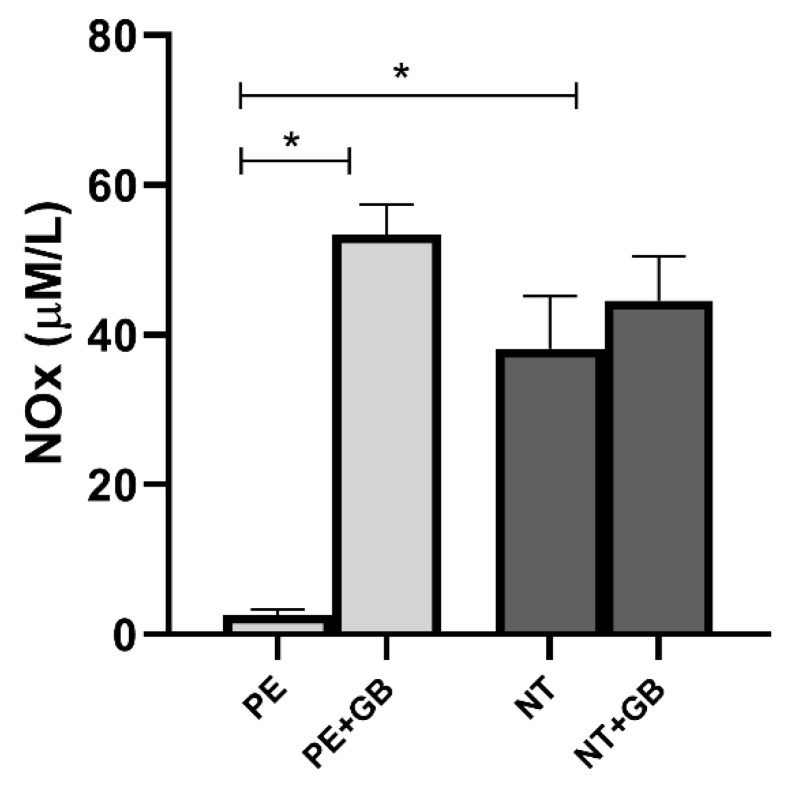
Nitrite + nitrate levels (Nox) in the cell supernatant of human umbilical vein endothelial cells (HUVEC) incubated for 24 h with 20% (*v*/*v*) plasma pool from NT and PE pregnant women and GB. Values are represented as means ± SEM. Comparisons between groups were assessed by One-Way ANOVA followed by Tukey’s Test. * (*p* < 0.05).

Nitrite and nitrate levels in supernatant from endothelial cells incubated with a pool of PE plasma presented lower levels of NOx compared with NT-plasma-treated cells (Figure 3). In this group, GB treatment increased NOx levels (*p* < 0.05). Comparison between NOx levels produced in HUVEC supernatant and HUVEC+GB also showed an increase in cells treated with GB (*p* < 0.05).

### 3.4. ROS Levels Are Elevated in Cells Treated with Plasma from NT, and GB Can Reduce Oxidative Stress in Cells Incubated for 30 min with PE Plasma

**Figure 4:**
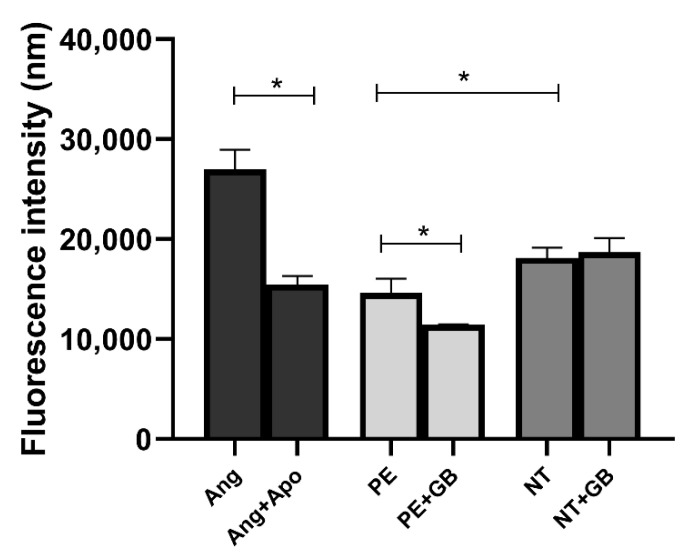
Intracellular reactive oxygen species (ROS) evaluated in 30 min by fluorescence of 2,7-dichlorodihydrofluorescein diacetate (DCFH) in the supernatant of human umbilical vein endothelial cells (HUVECs) incubated with 400 µM of angiotensin II (Ang), 100 µM of apocynin (Apo), 20% (*v*/*v*) pooled plasma from NT (n = 10) and PE (n = 10) (a) and GB (100 µM) for 24 h. Data are reported as means ± SEM. Comparisons between groups were assessed by One-Way ANOVA followed by Tukey’s Test. * (*p* < 0.05).

HUVECs incubated with angiotensin II (Ang) (an NADPH oxidase activator) showed higher levels of ROS (*p* < 0.05) in 30 min of incubation compared to cells incubated with angiotensin II plus apocynin (Apo—an antioxidant agent). The cells incubated with NT plasma showed higher fluorescence intensity than the PE group, while HUVECs incubated with GB showed lower levels of ROS (*p* < 0.05) at 30 min in the PE+GB group (Figure 4).

### 3.5. Supernatant Levels of MDA Indicate a Decrease in Lipid Peroxidation in GB-Treated Cells of the PE Group

**Figure 5:**
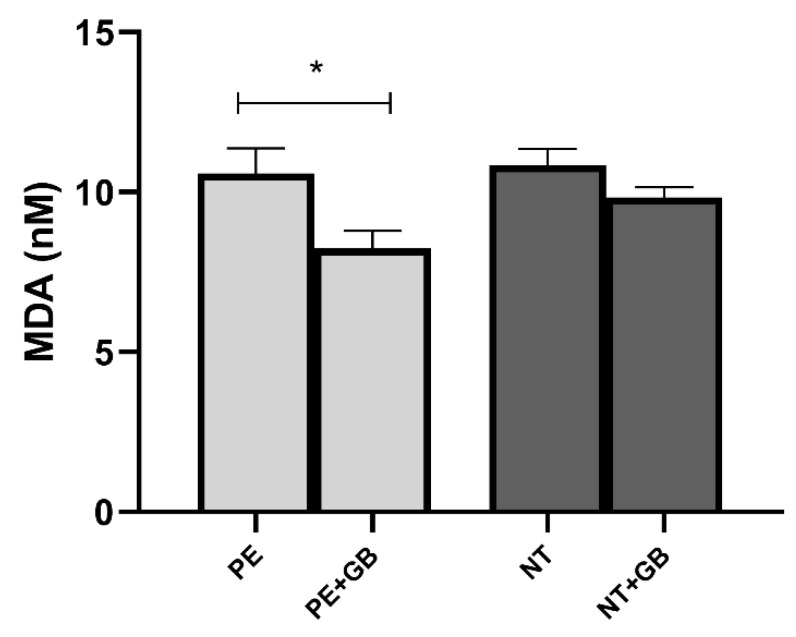
MDA was evaluated in the supernatant of human umbilical vein endothelial cells (HUVECs) incubated with 20% (*v*/*v*) pooled plasma from PE (n = 10) and NT (n = 10) and GB (100 µM) for 24 h. Data are reported as means ± SEM. Comparisons between groups were assessed by One-Way ANOVA followed by Tukey’s Test. * (*p* < 0.05).

The cells incubated with GB showed lower levels of MDA in the supernatant (*p* < 0.05) when compared with cultures with only pooled PE plasma (Figure 5). There was no difference between MDA measured in supernatants of HUVEC cultured with PE and NT.

### 3.6. Plasma from Pregnant Women did Not Differ in FRAP Activity, but Supernatant from PE Women Showed Higher Antioxidant Power, while GB Decreased this Response

**Figure 6:**
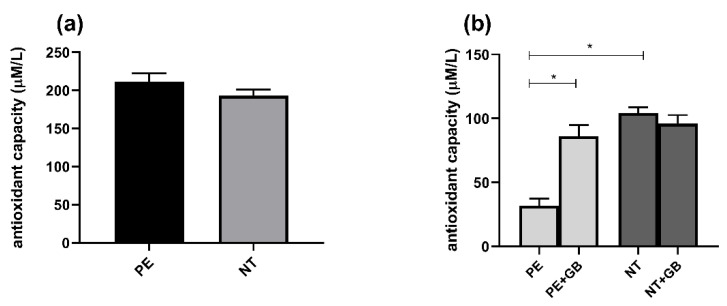
Ferric-reducing antioxidant power (FRAP) activity in plasma from PE and NT pregnant women (**a**) and in the supernatant of HUVEC culture with plasma and GB (**b**). Three independent experiments with replicates of 5 per group in each experiment. Data are presented as mean ± SEM. Mann–Whitney U test (**a**) and (ANOVA) followed by the Tukey’s Test (**b**). * (*p* < 0.05).

The FRAP values in plasma from pregnant women with PE and NT did not show significant differences (Figure 6a), but, when incubated with PE plasma, HUVEC showed higher antioxidant capacity compared to NT, and the addition of GB decreased these levels (Figure 6b).

**Figure 7:**
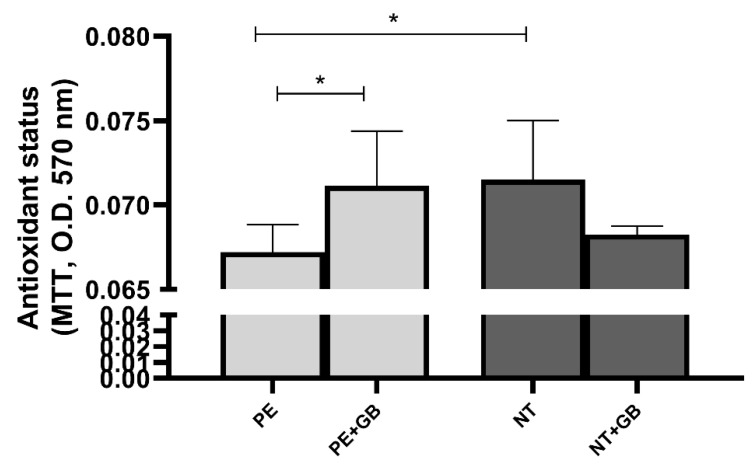
MTT reduction in the supernatant of HUVEC culture with plasma and GB. Three independent experiments with replicates of 5 per group in each experiment. Data are presented as mean ± SEM. (ANOVA) followed by Tukey’s test. * (*p* < 0.05).

Conversely, significant increases in antioxidant status were observed in these groups (NT and PE+GB) compared to PE through the MTT assay (Figure 7).

## 4. Discussion

Taken together, the results in this study demonstrated that GB incubation increased NO generation and NOx levels in HUVEC cultured with PE and NT plasma. Still, this drug decreased ROS levels and lipid peroxidation in cells incubated with PE plasma, and in the meantime, also increased the antioxidant capacity of these cells cultured with PE plasma. To our knowledge, this is the first study showing GB effects in HUVEC incubated with plasma from PE acting as an antioxidant drug.

It is already well-established in the literature that the plasma of normotensive pregnant women presents higher levels of NO and its metabolites. This molecule acts as key signaling in the cardiovascular system, controlling vascular tone and other important processes [24]. Most important, in pregnancy, NO participates actively in trophoblast invasion and placental development, being considered an essential vasodilator [25]. Alterations in the NO system, aggregated with an imbalance in antioxidant defense, contribute to endothelial dysfunction in PE women [26]. Furthermore, high levels of ROS formation can also contribute to impairing endothelial nitric oxide synthase (eNOS) function [27] and consequently decrease the bioavailability of NO.

We also observed higher levels of ROS in cells cultured with NT plasma compared to cells incubated with PE plasma. Toescu et al. showed that late pregnancy was associated with the formation of oxidizable particles (high LDL levels) and an increase in oxidative damage [28]. It is also important to highlight that during late pregnancy, an increase in basic metabolism and consumption of oxygen occurs [29]. In our work, the antioxidant capacity is also reduced in supernatants from cells cultured with NT plasma. Data from the literature indicate that total antioxidant capacity (TAC) is because of the decreased levels of serum albumin, bilirubin, and vitamin E [30].

The fact that ROS levels were low in cells incubated with PE plasma is associated with the results of the high antioxidant capacity found in the supernatant of these cells. This could be a compensatory mechanism in which cells are dedicated to fighting against ROS production and exhibiting a greater antioxidant capacity. Our group has already shown this antioxidant compensatory mechanism observed in hypertensive disorders of pregnancy, which matches this new finding. The NOx levels remain reduced in supernatants from cells incubated with PE to NT, also suggesting that other mechanisms may be involved in the NO bioavailability [31].

Treatment with GB decreases the antioxidant capacity, as this drug already has known antioxidant effects [32,33], mainly in diabetes. Likewise, when HUVECs were incubated with GB and PE plasma, they decreased ROS production. The role of glibenclamide as an antioxidant has been studied mainly in diabetes, since this drug is widely used for the treatment of this disease. Its role as an antioxidant has been explored in endothelial cells since 1998, when Okayama et al. [34] showed that this drug was able to decrease the adhesion of neutrophils induced by NO and hydrogen peroxide. Likewise, Murugan et al. [35] demonstrated that the conjunct treatment of GB and sialic acid decreased the production of superoxide anion and ROS, as well as reversed the low levels of NO in HUVEC. The same group also demonstrated that the co-treatment increased NO levels in the aorta of rats and reversed the impaired relaxation induced by acetylcholine in these animals. Also in an animal model, Geng et al. demonstrated that GB blocked the effects of hydrogen sulfide on eNOS activity [36].

A recent meta-analysis evaluated the safety and effectiveness GDM drugs treatment in 26 randomized controlled trials (RCTs) involving almost 5000 patients. GB is still behind metformin and insulin, and the authors stated that more RCTs are needed to verify its safety [37]. Furthermore, other authors have been discussing the dosage and safety of using GB in pregnancy. Like many medications used by pregnant women, adequate pharmacokinetic and pharmacodynamic data in pregnancy have been sorely lacking. Caritis et al. showed new dosages for women with GDM, but additional research is required to determine whether higher dosages can or should be used because GB crosses the placenta, and this should be taken into consideration for the pros and cons when considering the use of new drugs for pregnancy-related disorders [38]. Moretti and collaborators also showed no increased perinatal risks with GB [39]. Besides that, most trials often include few women and report few outcomes. Large, well-designed, and well-conducted trials are needed to better evaluate the effects of GB in pregnancy and PE.

As we have shown here in this study, GB can also act in an antioxidant capacity. The literature has been showing the role of GB, mainly in animal models, and the reports are still contrasting. Some studies have shown that GB increased total antioxidant capacity and decreased lipid peroxidation in the liver of rats, increasing the antioxidant superoxide dismutase and catalase [40], as well as malondialdehyde levels [41]. Recently, Alabi et al. demonstrated that in diabetes-induced rats, administered GB decreased catalase and nuclear factor kappa B [42], and Qasen et al. also demonstrated an increase in total protein levels in GB-treated rats [43]. Additionally, in a study using the HaCaT cell line, Klein and colleagues showed that GB inhibits cell migration [44], while other studies showed that GB co-treatment with metformin and irbesartan improves endothelial dysfunction and the cell migration capacity in HUVEC [45].

## 5. Conclusions

The GB treatment exerted a positive effect on the NO production by HUVEC incubated with plasma from NT and PE pregnant women, as well as in the reduction in oxidative stress. These results imply that GB has antioxidant effects on endothelial cells, and we believe in the future, it could be an alternative for the management of PE and, consequently, reduce the repercussions of the disease in patients. One of the limitations of the study may be related to the gestational age at sampling. However, we used a pool of plasma from PE and NT pregnant women, and we believe that the small difference between the groups is diluted within this sample. In Botucatu’s clinical service, patients with PE usually have their delivery resolved before, and normotensive patients come to the service shortly before delivery, a fact that may also contribute to the differences in the collection date.

## Figures and Tables

**Table 1:** Clinical, demographic, and biochemical characteristics of study subjects.

Parameters	Normotensive Pregnant	Preeclampsia	*P*
	(*n* = 10)	(*n* = 10)	(<0.05)
**Age (years)**	26.72 ± 0.71	27.32 ± 0.58	0.5177
**Race Caucasian**	8	9	>0.9999
**Non-caucasian**	2	1	>0.9999
**GAS (weeks)**	34.33 ± 0.35	31.87 ± 0.52	**0.0001**
**SBP (mm/Hg)**	115.70 ± 0.70	155.80 ± 0.20	**<0.0001**
**DPB (mm/Hg)**	73.30 ± 0.10	103.30 ± 0.20	**<0.0001**
**24-h Pr (mg)**	ND	1857 ± 539.40	-
**Uric acid (mg/dL)**	ND	5.15 ± 0.20	-

Abbreviations: GAS: gestational age at sampling; SBP: systolic blood pressure DBP: diastolic blood pressure; 24 h Pr: 24 h proteinuria; ND: not determined. Values are expressed as mean ± SEM. *p* < 0.05 vs. NT. Bold values are significant. Mann–Whitney U test.

## Data Availability

The data are contained within the article.
